# Association of trimester-specific gestational weight gain with child BMI by maternal BMI categories

**DOI:** 10.21203/rs.3.rs-6263508/v1

**Published:** 2025-04-01

**Authors:** Erin LeBlanc, Rachel Springer, Natalie Rosenquist, Anna Booman, Kimberly Vesco, Evelyn Sun, Byron Foster, Janne Boone-Heinonen

## Abstract

**Background/Objectives:** Information is lacking about how trimester-specific gestational weight gain (GWG) is associated with childhood body mass index (BMI) across maternal BMI categories. **Subjects/Methods:** We examined the association between GWG and child BMI in patients served by a national network of community health care organizations. We stratified by pre-pregnancy BMI (n=5721 normal weight; 5667 overweight; 3213 obesity class I; 1344 class II; 692 class III). Child BMI z-score and overweight and obesity status at age 5 were modeled as a function of total GWG and GWG rate in each trimester, controlling for GWG rate in previous trimester(s) and maternal characteristics, using modified Poisson regression. **Results:** Higher total GWG during pregnancy was positively associated with child BMI at 5 years of age in a linear, dose-dependent manner. When examined by trimester of pregnancy, a 1 kg/week in the first trimester was associated with a 0.24 to 0.42 increase in child BMI z-score. The same increase in the second trimester was associated with a 0.30 to 0.53 increase in child BMI z-score, although the associations were not significant in class II and III obesity classes. Associations between GWG in the third trimester and child BMI z-score were weak (0.12 to 0.21 increase in BMI z-score per kg/week increase in maternal weight) and not significant. **Conclusion:** Among a diverse and underserved pregnant population, GWG in the 1 ^st^ and 2 ^nd^ trimesters are most strongly associated with child BMI at age 5. Early pregnancy and mid-pregnancy may be key times to intervene to prevent overweight and obesity in offspring.

## Introduction

Obesity affects 20% of children aged 2 to 19 years in the US.[1] Childhood obesity often translates into obesity across the lifespan,[2–5] increasing the risk for diabetes, heart disease, and premature morbidity in adulthood.[6,7] Thus, obesity prevention in childhood is critical to addressing the obesity crisis and reducing chronic illness burden. Efforts to prevent obesity in childhood have had mixed success, with the most promising existing interventions beginning in early infancy.[8] However, risk conditions for obesity may begin even prior to birth: *in utero* exposure to overnutrition and insulin resistance as well as epigenetic changes during pregnancy, particularly in the 1st trimester, could contribute to childhood obesity risk.[9] Therefore, there is growing attention to the impact that the intrauterine environment during pregnancy has on obesity risk, with the hope that obesity prevention efforts that start before birth may be highly effective.

Both the pregnant parent's body mass index (BMI) at pregnancy onset and their gestational weight gain (GWG) during pregnancy contribute to the intrauterine environment and likely contribute to children's obesity risk.[10] While some obesity concordance between parents and offspring may be due to genetic and postnatal factors,[11] animal models that employ a common genetic background and carefully controlled environments during and after pregnancy have confirmed that maternal weight before and during pregnancy independently influence the body weight and adiposity of offspring long-term.[12]

Given the key role that both BMI at pregnancy onset and GWG play in the health of the intrauterine environment in which the fetus is developing, the National Academy of Medicine (NAM) recommends targets for GWG based on BMI categories.[13] However, due to lack of data in pregnant women with pre-pregnancy BMI ≥ 35 $kg/m^2$,[14–17] the NAM BMI categories only include categories for underweight (< 18.5), normal (18.5-<25), overweight (25-<30), and obesity (≥ 30), and do not address GWG for women with severe obesity (Class II-III, BMI ≥ 35). Data are also lacking on the impacts of BMI at pregnancy onset and GWG on child obesity outcomes for racial and ethnic minority groups, despite these populations having the highest risks of severe maternal obesity and childhood obesity.[18,19] In addition, the few studies from the United States (U.S.) that examine the impacts of GWG on offspring in pregnant women with severe obesity rely predominantly on data from birth records,[14] and lack the longitudinal measures required to examine the trimester-specific impact of GWG on child growth.

While optimizing GWG is a promising approach for mitigating child obesity risk, more data are needed regarding optimal GWG across all pre-pregnancy BMI categories, particularly according to trimester of pregnancy, given that the metabolic impact of the intrauterine environment may vary by trimester.[20] This study examines associations between trimester-specific GWG and childhood BMI at age 5, with an emphasis on examining parents with high pre-pregnancy BMI. We focus on the first 2,000 days (conception to 5 years of age) because growth during this time covers the period of early adiposity rebound and early life development of health behaviors.[21] We hypothesized that GWG, especially in the 1st trimester,[9] would be associated with child BMI z-score at age 5 across all BMI categories, including among those with severe obesity.

### Materials/Subjects and Methods

## Study Population

The PReventing Obesity through healthy Maternal gestational weight gain In the Safety nEt (PROMISE) Study cohort is derived from electronic health record (EHR) data from the Accelerating Data Value Across a National Community Health Center Network (ADVANCE) Clinical Research Network (CRN), a member of PCORnet®. ADVANCE is a multicenter collaborative led by OCHIN in partnership with Fenway Health, Health Choice Network, Oregon Health & Science University, and University of Washington. OCHIN-affiliated community-based healthcare organizations (CHCOs) serve patients regardless of insurance coverage or ability to pay, including uninsured and under-insured patients, undocumented immigrants, and members of other marginalized groups. The PROMISE study was approved by the Oregon Health & Science University Institutional Review Board.

The PROMISE cohort includes 77,599 pregnancies (among 65,179 individuals) with pregnancy onset dates between 4/16/2004 and 7/6/2020. Patients included in the cohort meet the following criteria: age 15 years or older at pregnancy onset, 20–42 weeks gestational age at delivery, and the following data available in the EHR: adult height, pre-pregnancy weight, ≥ 1 weight measure in the 2nd or 3rd trimester, and ≥ 1 additional pregnancy weight measure.

### Study variables

**Pregnancy weights (exposure).** Weights during pregnancy were extracted from the clinical record and implausible measures were excluded as previously described.[22] Weights were classified into trimesters (0 to < 14 weeks, 14 to < 28 weeks, and 28 weeks to end of pregnancy).

**Child BMI-z score at age 5 (outcome).** After extracting and cleaning child weights and heights using the R package growthcleanr,[23] we ascertained BMI from same-day height and weight recorded closest to 60 months of age, among measures from 48–72 months, then calculated BMI z-score at age 5 using the cdcanthro package in R, which generates z-scores and percentiles based on the 2000 Centers for Disease Control and Prevention (CDC) extended BMI growth charts.[24] For secondary analysis, we classified child BMI as overweight (> 85th percentile) or not, as well as obese (≥ 95th percentile) or not.

**Maternal pre-pregnancy BMI (effect measure modifier).** Pre-pregnancy BMI was calculated using the median height among all measures recorded after age 16 years and the weight measurement closest to pregnancy onset date within one year (365 days) prior to and 14 weeks (97 days) after pregnancy onset date or, if no measurement fit that criterion, patient-reported pregravid weight (n = 7,869, 11%). *Pre-pregnancy BMI* was then categorized as normal (18.5 to < 25 kg/m$^2$), overweight (25 to < 30 kg/m$^2$), obesity class I (30 to < 35 kg/m$^2$), obesity class II (35 to < 40 kg/m$^2$), and obesity class III (≥ 40 kg/m$^2$).

**Confounders.** A full description of how the confounding variables were derived from EHR data has been previously described.[22] For this analysis, confounders included maternal age, tobacco use (current, former, never, unknown), and income level (0–50%, 51–100%, 101–200%, > 200%, or unknown) at the pregnancy onset date.[22] Payer type (public insurance, private insurance, other non-comprehensive insurance, or uninsured) was defined as the most frequent payer type recorded from the 2nd trimester through delivery.[25] Race and ethnicity were categorized as Hispanic, non-Hispanic (NH) American Indian/Alaska Native, NH Asian, NH Black, NH Native Hawaiian/Other Pacific Islander, NH Other/Multiple races, NH White, and unknown race/ethnicity;[26] preferred spoken language as English, Spanish, other, or unknown; and state of residence as Oregon, Washington, California, or other.

**Variables used for sensitivity analysis**. We collected patient history of pre-pregnancy diabetes mellitus (DM) or gestational diabetes (GDM) using ICD 9/10 codes in encounters or problem lists as previously described.[22] For pre-pregnancy hypertension and hypertensive disorders of pregnancy, we used International Classification of Diseases (ICD) 9/10 in encounters or problem lists as well as evidence of sustained elevated blood pressure in the clinical record.[22] Preterm birth was defined as gestational age (GA) < 37 completed weeks at delivery.

## Analytic inclusion criteria

**Main analysis.** For the analysis of rate of GWG, we required known, plausible birthweight singleton pregnancies, live births, and ≥ 2 maternal weight measures during pregnancy that were ≥ 2 weeks apart (Fig. 1). Plausible birthweight was identified based on gestational age and sex-specific z-scores.[27] As distinct considerations apply to those with underweight at pregnancy onset,[28,29] we excluded pregnancies with pre-pregnancy BMI < 18.5 kg/m.[2] Finally, we excluded those with implausible GWG rates in any trimester and required that the child associated with each pregnancy have at least 1 same-day height and weight measure between 48 and 72 months. For the analysis of total GWG, we further required the pregnancy to have resulted in a full-term delivery.

**Sensitivity analyses**. For sensitivity analyses for GWG (both trimester specific and total GWG), we excluded pregnancies with pre-existing hypertension or HDP (**Supplemental Fig. 1a**) and, separately, excluded pregnancies with pre-existing diabetes or GDM (**Supplemental Fig. 1b**).

### Statistical analysis

All analyses were stratified by pre-pregnancy BMI category. We first compared distributions of clinical and sociodemographic characteristics across pre-pregnancy BMI categories, evaluating magnitude and direction of differences between BMI groups by calculating standard mean differences (SMD > 0.2 indicating meaningful differences between groups).

*GWG rate and total GWG estimation*. We estimated weight change rate (kg/week) within each trimester using linear piecewise latent growth curve models (MPlus, 8th ed).[30,31] We excluded implausible GWG rates (+/- 4 median absolute deviations;[32] <1% of pregnancies) in order to stabilize association model estimates. Total GWG was calculated for term pregnancies; we estimated weight at delivery and weight at conception based on observed weight measures using cubic spline regression (Stata v17); total GWG was calculated as the difference between two quantities.

*Associations between GWG and child BMI-z-score at age 5*. For each pre-pregnancy BMI group and trimester (1, 2, 3, or total GWG), we fit a series of linear regression analyses (R version 4.4.0)[33] modeling child BMI z-score at age 5 years as a function of GWG and maternal confounders (see above). Trimester 2 and 3 models also adjusted for GWG in previous trimester(s) to recognize their temporal sequence and to align with prior studies.[20,34–37]

First, we fit crude (unadjusted) models containing GWG rate (kg/week) of a single trimester or total GWG (kg). Based on graphical display and statistical testing of higher order terms, we determined that associations were linear. Second, we fit primary models adjusting for confounders, which were selected[38] based on our a priori conceptual framework described in a previous publication:[22] baseline maternal characteristics (continuous BMI, age, race and ethnicity, language, state of residence), pregnancy characteristics theorized to not be influenced by GWG (early pregnancy tobacco use, income, payer type), and, for trimester 2 and 3, GWG rate in the previous trimester(s). To facilitate interpretation, we also fit analogous logistic regression models for child overweight (vs normal weight) and obesity (vs normal or overweight) status.

## RESULTS

### Demographics

The study population included 16,637 mother-child dyads from the US, about two-thirds of whom identified as Hispanic and over half of whom reported preferred spoken language other than English (Table 1 **and Fig. 1**).

The majority had household incomes below the federal poverty line, and over 80% had Medicaid. The population was nearly evenly split among those with pre-pregnancy normal weight (34%), overweight (34%), and obesity (32%). Of those with pre-pregnancy obesity, 61% met class I obesity criteria, 26% met class II criteria, and 13% met class III criteria. Maternal age, state of residence, insurance payer type, and tobacco use did not differ across BMI categories. NH White race was overrepresented in normal weight and obesity class III; NH Black race in obesity class III; NH Asian race in normal weight; and Hispanic ethnicity in overweight and obesity class I and II. Compared to lower BMI categories, those with class III obesity were more likely to speak English and less likely to speak Spanish. As expected, the number of pregnant women excluded for preexisting diabetes and hypertension in sensitivity analyses was greater in the higher BMI categories, but the exclusions did not impact associations between obesity classes and sociodemographic characteristics (data not shown).

Table 1
Baseline characteristics by obesity class

| | | All weight classes | Normal | Overweight | Obesity Class 1 | Obesity Class 2 | Obesity Class 3 | SMD |
|---|---|---|---|---|---|---|---|---|
| | **N** | **16,637** | **5721** | **5667** | **3213** | **1344** | **692** | |
| Age, mean (SD) | | 27.97 (5.99) | 26.67 (5.97) | 28.26 (5.88) | 29.12 (5.99) | 28.94 (5.74) | 29.10 (5.65) | 0.196 |
| Race/ethnicity (%) | NH White | 18.2 | 24.2 | 14.8 | 13.4 | 17.0 | 19.7 | 0.319 |
| | NH Black | 7.4 | 7.1 | 6.3 | 7.9 | 8.0 | 15.6 | |
| | NH Asian | 3.6 | 6.8 | 2.7 | 1.5 | 1.1 | < 1 | |
| | NH NH/OPI | 0.4 | 0.3 | 0.3 | 0.4 | 0.8 | < 1 | |
| | NH AI/AN | 0.3 | <1 | 0.3 | 0.5 | < 1 | < 1 | |
| | NH Other or Multiple | 0.7 | 0.9 | 0.4 | 0.7 | 0.9 | < 1 | |
| | Hispanic | 67.9 | 58.7 | 73.8 | 74.6 | 70.0 | 60.5 | |
| | Unknown | 1.5 | 1.9 | 1.3 | 1.0 | 1.7 | 1.4 | |
| Language (%) | English | 42.9 | 48.5 | 35.7 | 38.0 | 49.6 | 65.3 | 0.345 |
| | Spanish | 51.5 | 43.4 | 59.3 | 58.0 | 47.6 | 33.5 | |
| | Other | 5.6 | 8.1 | 5.0 | 4.0 | 2.8 | < 1 | |
| State (%) | OR | 36.7 | 35.6 | 36.3 | 38.3 | 40.8 | 32.5 | 0.129 |
| | CA | 46.3 | 46.5 | 48.4 | 44.6 | 42.3 | 44.1 | |
| | WA | 2.7 | 2.8 | 2.6 | 2.9 | 1.9 | 3.6 | |
| | Other | 14.3 | 15.1 | 12.7 | 14.2 | 15.0 | 19.8 | |
| FPL (%) | 0–50% | 23.9 | 25.6 | 22.2 | 23.0 | 22.8 | 30.5 | 0.139 |
| | > 50–100% | 24.2 | 21.6 | 24.5 | 26.8 | 27.4 | 24.1 | |
| | > 100–200% | 20.7 | 20.3 | 21.2 | 21.2 | 20.6 | 17.6 | |
| | > 200% | 6.5 | 8.1 | 6.0 | 5.4 | 5.7 | 4.3 | |
| | Unknown | 24.7 | 24.4 | 26.1 | 23.6 | 23.4 | 23.4 | |

Percentages based on cell sizes < 10 are reported as < 1%

| | | All weight classes | Normal | Overweight | Obesity Class 1 | Obesity Class 2 | Obesity Class 3 | SMD |
|---|---|---|---|---|---|---|---|---|
| | **N** | **16,637** | **5721** | **5667** | **3213** | **1344** | **692** | |
| Payer (%) | Medicaid | 84.2 | 81.5 | 85.0 | 86.4 | 85.6 | 87.6 | 0.128 |
| | Medicare or Other Public | 0.5 | 0.5 | 0.5 | 0.5 | < 1 | < 1 | |
| | Private | 8.3 | 11.7 | 7.1 | 5.9 | 6.5 | 6.2 | |
| | Uninsured | 6.9 | 6.3 | 7.4 | 7.0 | 7.4 | 4.9 | |
| | Unknown | 0.1 | < 1 | < 1 | < 1 | < 1 | < 1 | |
| Tobacco use (%) | Current | 6.9 | 7.3 | 5.5 | 6.2 | 9.4 | 13.2 | 0.186 |
| | Former | 7.6 | 8.3 | 6.4 | 7.3 | 7.9 | 12.4 | |
| | Never | 51.4 | 48.1 | 53.9 | 54.3 | 50.0 | 46.2 | |
| | Unknown | 34.1 | 36.4 | 34.1 | 32.2 | 32.7 | 28.2 | |
| Gestational age, weeks, mean (SD) | | 39.29 (1.58) | 39.38 (1.52) | 39.30 (1.57) | 39.22 (1.64) | 39.21 (1.64) | 38.97 (1.75) | 0.11 |
| Percentages based on cell sizes < 10 are reported as < 1% | | | | | | | | |

### Rates of GWG in each trimester

In the 1st trimester, the majority of women in the overweight, obesity I, obesity II, and obesity III classes experienced weight loss (median of -0.03 to -0.13 kg/week), with the amount of weight loss increasing with increasing baseline BMI (Fig. 2). In contrast, the majority of women with normal weight experienced some weight gain (median of 0.04 kg/week). In the 2nd and 3rd trimesters, most women across all BMI classes experienced weight gain, with rate of gain being lower in the higher BMI categories, and lowest among those in obesity class III (2nd trimester: median kg/week 0.35 to 0.21 kg/week across obesity class I to class III). Compared to the 2nd trimester, GWG was higher in the 3rd trimester for nearly all BMI categories (median 0.44 to 0.38 kg/week across class I to III) although those in the normal BMI category had slightly lower median GWG in the 3rd trimester (0.51kg/week) than in the 2nd trimester (0.53 kg/week).

### Association between Trimester-Specific GWG and child BMI z-score at age 5

In the primary adjusted models, a more positive rate of GWG in the 1st trimester was associated with higher child BMI z-score at age 5 among all pre-pregnancy BMI categories (Fig. 3), with estimates ranging from an expected change in child BMI z-score of 0.24 per 1 kg/week unit increase (e.g., from − 2 to -1 kg/week or from

1 to 2 kg/week) in GWG (obesity class I, 95% confidence interval [CI]: 0.06, 0.43) to an expected change in z-score of 0.46 per 1 kg/week unit increase (obesity class II, 95% CI: 0.21, 0.72).

Second trimester GWG was also positively associated with child BMI z-score at age 5 across all pre-pregnancy BMI categories, with estimates of child BMI z-score increase per 1 kg/week unit increase in maternal weight ranging from 0.30 (normal, 95% CI: 0.09, 0.51) to 0.53 (overweight, 95% CI 0.34, 0.71), although the associations between child BMI and maternal GWG were not significant ($\alpha = 0.05$) in the class II and III obesity categories, where there were smaller sample sizes and thus less precision.

More positive GWG in the 3rd trimester was significantly associated with higher child BMI-z score among those in the pre-pregnancy overweight category (0.21 [95% CI: 0.05, 0.38] increase in BMI z-score per kg/week unit increase in maternal weight), associations were weaker and not significant in the 3rd trimester for the other pre-pregnancy BMI classes.

Sensitivity analyses revealed similar patterns and estimates when the following were excluded: those with pre-pregnancy hypertension and HDP (Supplement Fig. 2a); those with DM and GDM (Supplement Fig. 2b); and those with preterm births (data not shown).

## Association between total GWG and child BMI z-score at age 5

More positive total GWG was associated with higher child BMI z-score across all pre-pregnancy BMI categories (median estimates of increase in child BMI z-score per 1 kg unit increase in total GWG: 0.01 to 0.02), although the association was not significant in those with obesity class III in the primary analysis (Fig. 4). After excluding those with pre-pregnancy DM or GDM, the association between total GWG and 5-year BMI z-score was stronger and statistically significant in obesity class III (estimate of 0.03, 95% CI 0.01, 0.05) (**Supplemental Fig. 3a**). Estimates were not substantially impacted by removing pre-pregnancy hypertension and HDP (**Supplemental Fig. 3b**).

## Association between GWG and Child Having Overweight or Obesity at Age 5

Estimates of the relative odds of a child having overweight (BMI z-score > 85th percentile) at age 5 years per 1 kg/week unit of GWG in the 1st trimester ranged from 1.16 (95% CI 0.83, 1.61) in those with class I obesity to 1.97 (95% CI 1.25, 3.10) in those with class II obesity (Fig. 5a). Estimates based on GWG in the 2nd trimester were similar to those in the 1st trimester for those with normal weight and class III obesity (normal weight: 1.61 in 2nd trimester vs 1.69 in 1st trimester; obesity class III: 1.53 vs 1.69), but notably (though not significantly) higher for the overweight, obesity class I, and obesity class II categories (Overweight: 2.44, vs 1.90; Class I: 2.39 vs 1.60; Class II: 2.31 vs 1.97). Most 3rd trimester ORs were not significantly different from 1, except among those with overweight (OR 1.61, 95% CI: 1.18, 2.20). Similar findings were found for odds of obesity (BMI > 95) at age 5 years per 1 kg/week of GWG in each trimester (Fig. 6a).

When we compared those with class I, II, and III obesity who were at the 75th percentile of total GWG to those with total GWG in the 25th percentile, the relative odds of having a child with overweight (BMI > 85%) were 1.17 (95% CI: 1.07, 1.27) times higher for those with class I obesity and 1.24 (95% CI: 1.08, 1.42) times higher for those with class II obesity, with the odds not significantly different for those with class III obesity **(Fig. 5b)**. Similarly, the relative odds of having a child with obesity (> 95%) in those with 75th compared to 25th percentile total GWG were 1.18 (95% CI: 1.07, 1.30) times higher for those with class 1 obesity and 1.23 (95% CI: 1.06, 1.41) times higher in those with class II obesity, with the odds not significantly different for those with class III obesity **(Fig. 6b)**.

## Discussion

Among a diverse and systemically underserved pregnant population, higher total GWG during pregnancy was positively associated with child BMI at 5 years of age in a linear, dose-dependent manner. By analyzing GWG rate as a continuous variable, our study was able to quantify the full range of weight change within each trimester, which included weight loss in all trimesters and weight gain as high as 1 kg/week. The positive associations between total GWG and child BMI z-score appear to be driven mainly by weight change in the 1st and 2nd trimesters, rather than in the 3rd trimester. In the 3rd trimester, associations between GWG and child BMI z-scores were weak and mostly not significant. Similar patterns were found when examining the association between GWG and odds of children having overweight or obesity at age 5. Especially among pregnant persons with severe obesity, most of whom lost weight in the 1st trimester, those either less negative or more positive GWG in the 1st and 2nd trimesters, but not the 3rd trimester, had higher odds of having an offspring with BMI z-score > 85th percentile (overweight) or > 95th percentile (obesity) at age 5.

Associations between total GWG and child BMI have been previously observed.[39] However, few studies have examined the association between trimester-specific GWG and child BMI. In the 1st trimester, the maternal pre-pregnancy and early pregnancy metabolic milieu influence early placental function and gene expression, which could impact risk of obesity in childhood.[40] In the 2nd trimester, the placenta is maturing, so overnutrition during this time could negatively impact placental maturation and function. The associations between 3rd trimester GWG and child BMI may be weaker because placental and fetal development is further along, and because fluid shifts play a larger role in 3rd trimester weight change compared to earlier trimesters. Of those that have examined the association of early GWG and childhood obesity, results have been mixed, with some studies reporting that GWG in the 1st and 2nd trimesters were most associated with child BMI (consistent with our findings).[20,41,42] However, others reported significant associations only for 1st trimester GWG(ref), only mid-pregnancy or 2nd trimester GWG,[43] or for all three trimesters.[44] Notably, to date, no studies have reported an association between only 3rd trimester GWG and child BMI.

Variation in study findings may be due to differences in population baseline BMI: in our study, we found significant associations in all trimesters for individuals in the “overweight” BMI category, but only in the 1st trimester for those in obesity classes I and II. However, previous studies were mostly based on populations with middle to high socioeconomic status and low prevalence of severe obesity. Our work extends prior findings by examining the association between trimester-specific GWG and child obesity risk by pre-pregnancy BMI class, including among a sizable group of individuals with severe obesity. This study is also one of the few to focus on socially marginalized populations, who have higher rates of child obesity.[45] Psychosocial stress, environmental exposures, and other exposures that differentially impact socially marginalized populations can contribute to, magnify, or compete with GWG effects on childhood BMI, which make examining these associations in marginalized racial and ethnic groups, as well as those who have low or unstable incomes or who lack health insurance, of key importance for study in this area.[46,47]

This work is consistent with our previous findings that a higher rate of weight gain in pregnancy is associated with higher risk of large for gestational (LGA) and lower risk of small for gestational age (SGA) births across all pre-pregnancy BMI groups, with the strongest associations seen in the 1st and 2nd trimesters.[31]

These findings suggest several important topics for future research. First, investigating the association between 1st and 2nd trimester GWG and other child cardiovascular health markers, such as blood pressure and lipids, would help to better understand the association between trimester-specific GWG rate and long-term child health. However, the large confidence intervals in our study for the Class III obesity group demonstrate the need for more studies that focus on women who enter pregnancy with a high BMI. Finally, prospective studies that collect biological data across pregnancy and into the postpartum period can evaluate potential mechanisms of by which GWG in early and midpregnancy could affect child obesity risk. Such mechanisms could inform interventions that aim to optimize metabolic health in pregnancy.

These findings have several clinical implications. Early pregnancy GWG was more strongly associated with risk of childhood obesity compared to pregnancy GWG in the 3rd trimester. Accordingly, interventions that start early in pregnancy may be more likely to impact future childhood BMI. In addition, many women, especially those with more severe obesity, lost weight in the first trimester; early interventions could consider targeting those who are gaining weight in the first trimester.

Our study had several limitations. While GWG is routinely collected and provides a clinically useful tracking tool, weight is a mix of multiple components including the fetus, placenta, amniotic fluid, extracellular fluid, other tissue (fat), uterine/breast tissue, and blood. Our study could not differentiate weight gain associated with each of these components. Additionally, we acknowledge that trimesters are arbitrary time periods applied to processes that progress continuously, and reliance on these time points may explain some differences with prior work that used different time periods. We relied on medical record data, which is known to contain measurement error. To minimize this error, we used an outlier-screening algorithm designed for pregnancy weight.[22] BMI z-score may also not be as accurate among children with the most severe obesity.[48] Another limitation is that individuals with clinical risk factors (e.g., preexisting DM) may have been underrepresented in our sample as they were more likely to be referred to specialized care and not captured in the OCHIN database. Exposure to medications was also not captured. These limitations are balanced by the examination of an underserved study population with a relatively high prevalence of all three obesity classes at baseline, and with enough clinical data to examine trimester-specific GWG.

In conclusion, among a diverse and underserved pregnant population with a high prevalence of overweight and obesity, including severe obesity, GWG in the 1st and 2nd trimesters were most highly associated with child BMI and risk of overweight and obesity at age 5, which is a key age at which to predict long-term metabolic risks. These data indicate that early- and mid-pregnancy may be key times to intervene on GWG to prevent the development of overweight and obesity in offspring.

## Declarations

**Funding:** ADVANCE is funded through the Patient-Centered Outcomes Research Institute (PCORI), contract number RI-OCHIN-01-MC. This work was supported by the National Institute of Diabetes and Digestive and Kidney Diseases (R01DK118484). The funding bodies had no involvement in the design and collection, analysis, or interpretation of the data or in writing the manuscript.

**Acknowledgements**: We thank Neon Brooks for her assistance with editing and Jordan Hill for help with manuscript preparation. The research reported in this work was powered by PCORnet®. PCORnet has been developed with funding from the Patient-Centered Outcomes Research Institute® (PCORI®) and conducted with the Accelerating Data Value Across a National Community Health Center Network (ADVANCE) Clinical Research Network (CRN). ADVANCE is a Clinical Research Network in PCORnet® led by OCHIN in partnership with Health Choice Network, Fenway Health, University of Washington, and Oregon Health & Science University. ADVANCE's participation in PCORnet® is funded through the PCORI Award RI-OCHIN-01-MC.

**Author Contributions:** ESL played an important role in interpreting the results, drafted the manuscript, approved the final version, and agreed to be accountable for all aspects of the work in ensuring that questions related to the accuracy or integrity of any part of the work are appropriately investigated and resolved. RS analyzed data, played an important role in study design and interpreting the results, revised the manuscript, approved the final version, and agreed to be accountable for all aspects of the work in ensuring that questions related to the accuracy or integrity of any part of the work are appropriately investigated and resolved. NAR played an important role in study design and interpreting the results, revised the manuscript, approved the final version, agreed to be accountable for all aspects of the work in ensuring that questions related to the accuracy or integrity of any part of the work are appropriately investigated and resolved. AB played an important role in study design and interpreting the results, revised the manuscript, approved the final version, agreed to be accountable for all aspects of the work in ensuring that questions related to the accuracy or integrity of any part of the work are appropriately investigated and resolved. KKV played a role in conceiving the work that led to the submission, played an important role in interpreting the results, revised the manuscript, approved the final version, and agreed to be accountable for all aspects of the work in ensuring that questions related to the accuracy or integrity of any part of the work are appropriately investigated and resolved. ES played an important role in interpreting the results, revised the manuscript, approved the final version, and agreed to be accountable for all aspects of the work in ensuring that questions related to the accuracy or integrity of any part of the work are appropriately investigated and resolved. BAF played an important role in interpreting the results, revised the manuscript, approved the final version, and agreed to be accountable for all aspects of the work in ensuring that questions related to the accuracy or integrity of any part of the work are appropriately investigated and resolved. JBH conceived and designed the work that led to the submission, and played an important role in interpreting the results, revised the manuscript, approved the final version, and agreed to be accountable for all aspects of the work in ensuring that questions related to the accuracy or integrity of any part of the work are appropriately investigated and resolved.

**Competing Interests statement:** Dr. Vesco has received research from Pfizer Independent Grants for Learning and Change to develop a menopause education curriculum for residents, which is unrelated to the current work. The other authors have no disclosures.

**Data availability Statement:** Raw data underlying this article were generated from multiple health systems across the OCHIN network; restrictions apply to the availability and re-release of data under organizational agreements. Researchers interested in access the study data can find relevant information at https://ochin.org/research/

## Figures

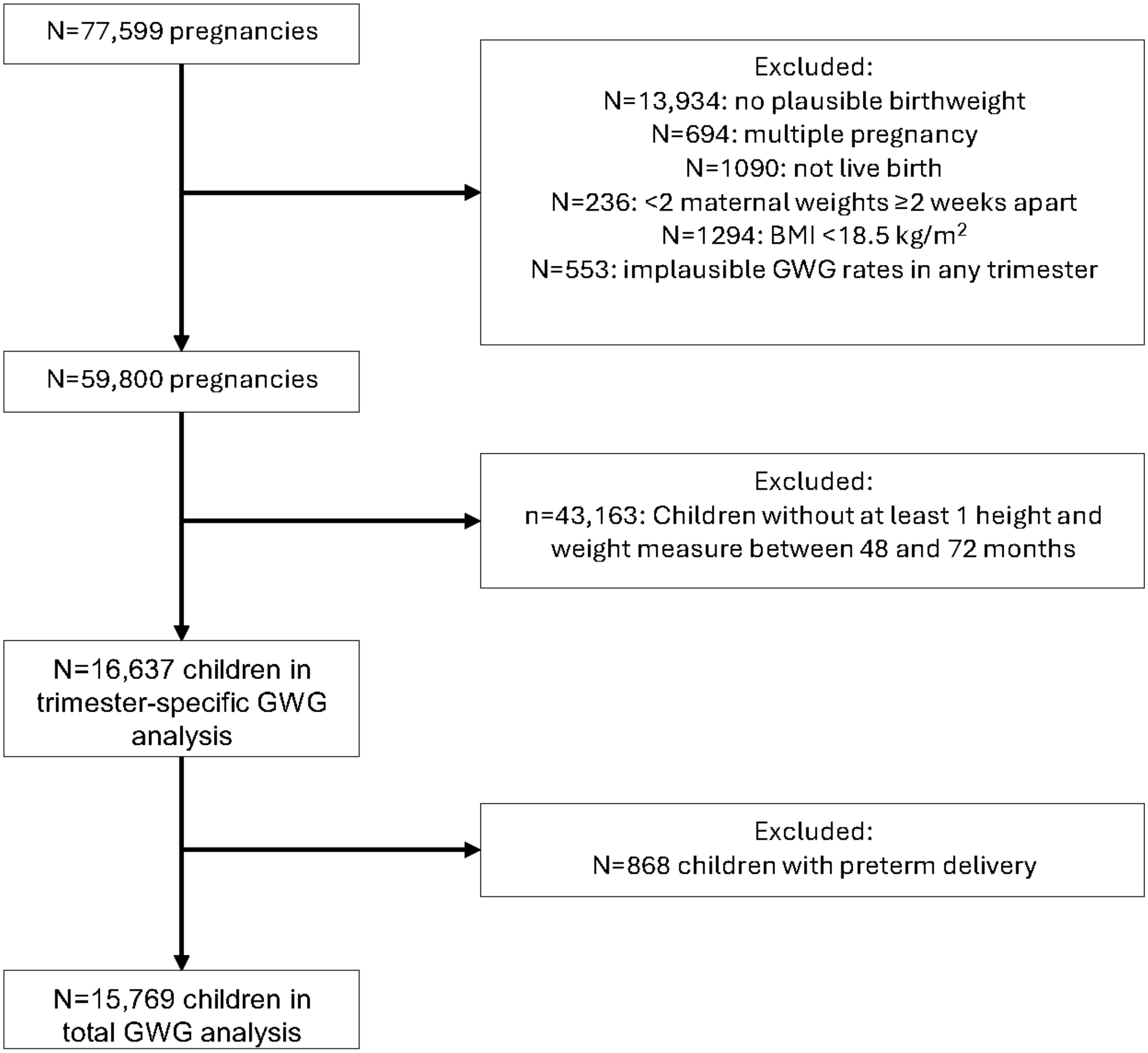

**Figure 1**

Consort diagram

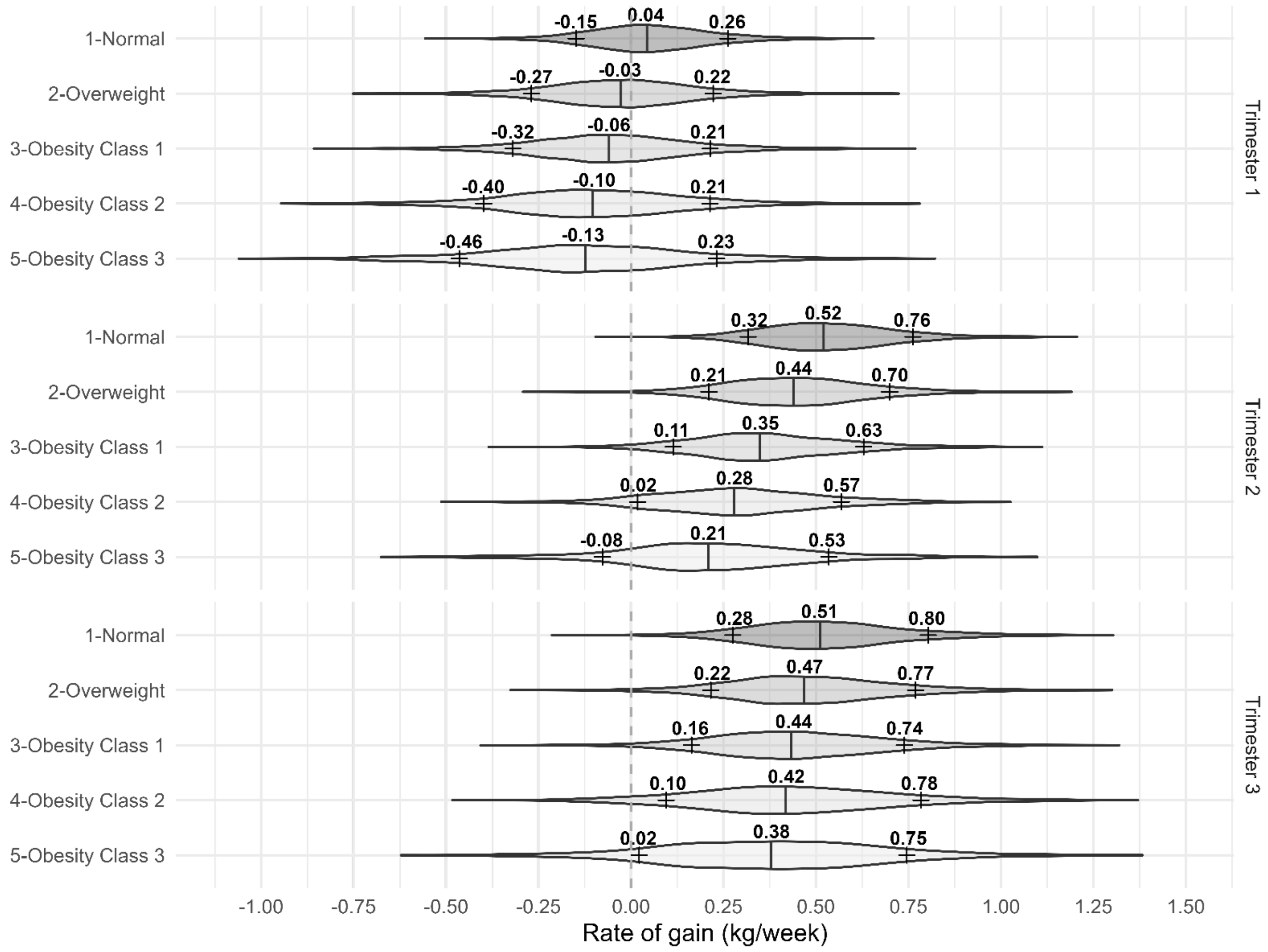

### Figure 2

Distribution of the rate of gestational weight change (median, 10th, and 90th percentiles*), by trimester and maternal pre-pregnancy BMI class

*The median is represented by the middle vertical line; the 10th and 90th percentiles are represented by the 1st and 3rd crosses, respectively; the length of each plot represents the full range of the observed data

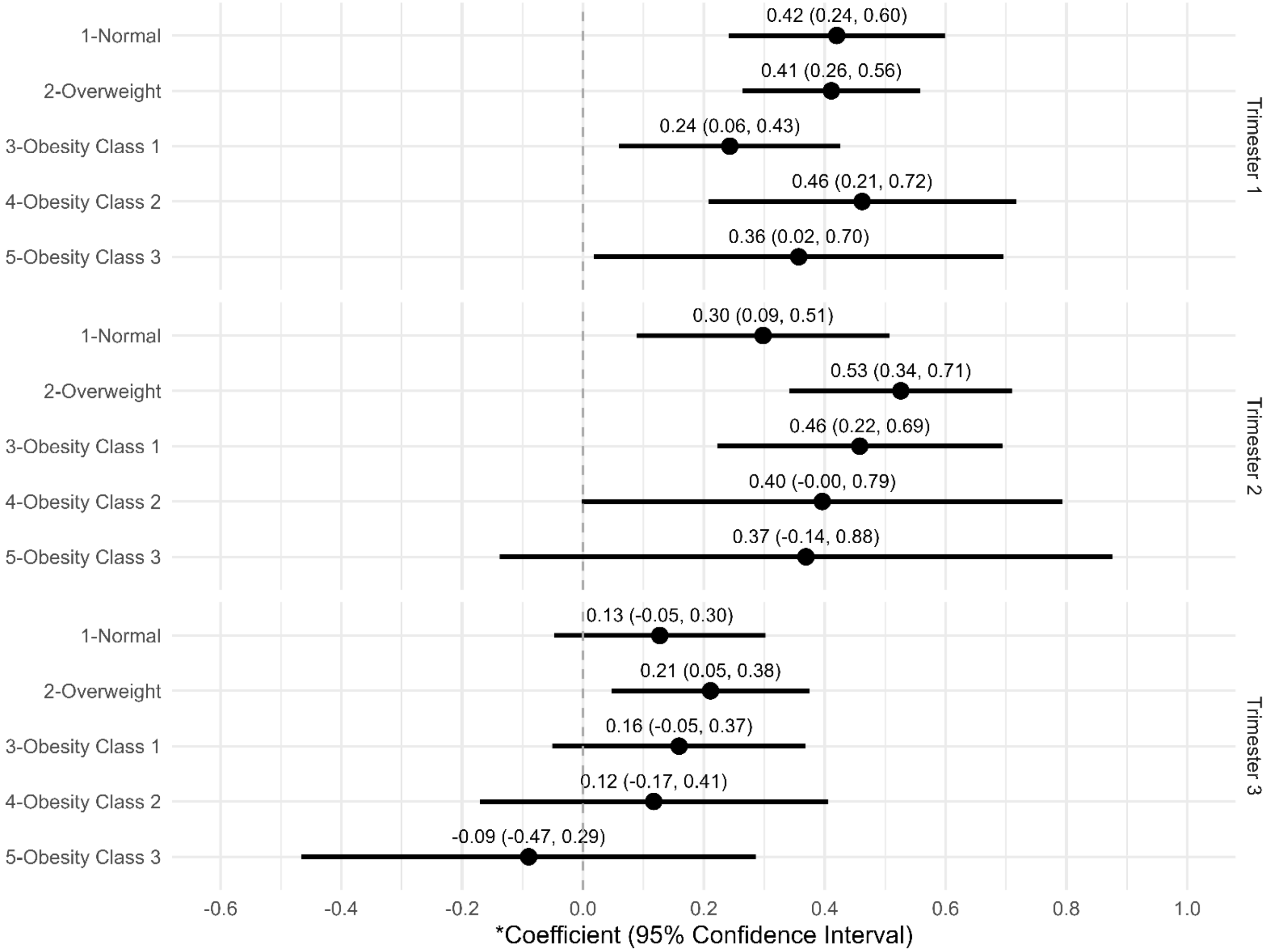

**Figure 3**

Association* of trimester-specific gestational weight gain rate (kg/week) with child BMI z-score at age 5, by trimester and maternal pre-pregnancy BMI class

*The coefficient represents the expected change in child BMI z-score associated with a 1kg/week unit increase in GWG (e.g., from 1 to 2 kg/week); analyses adjusted for previous trimester(s) GWG rate(s) and maternal characteristics

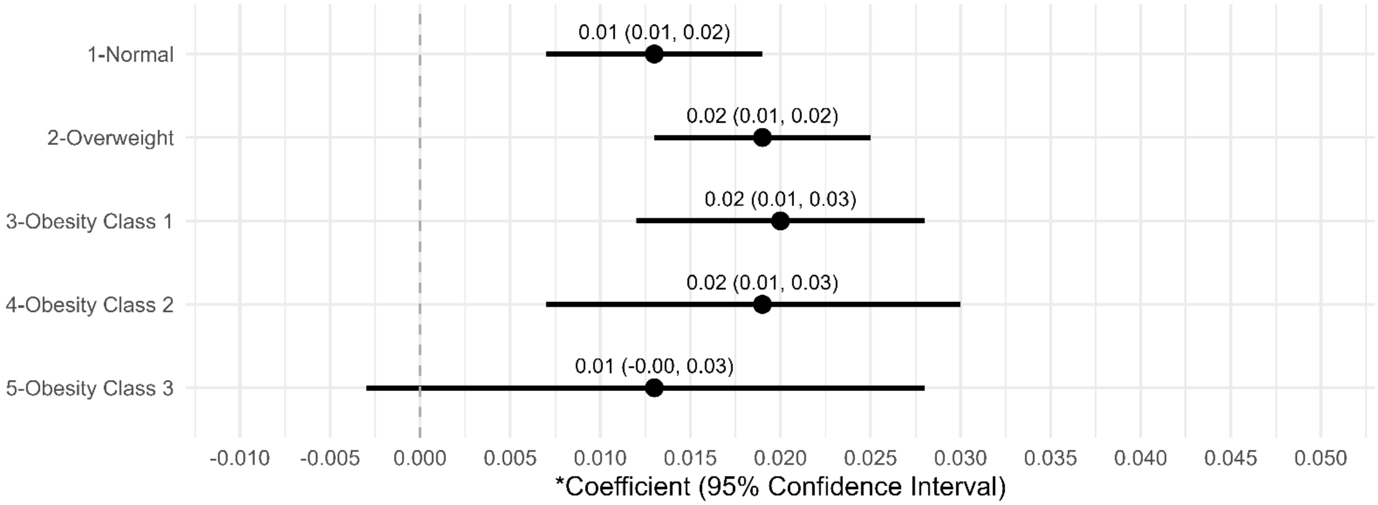

### Figure 4

Association* of total GWG (kg) with child BMI z-score at age 5, by maternal pre-pregnancy BMI class

*The coefficient represents the expected change in child BMI z-score associated with a 1kg unit increase in total GWG (e.g., from 20 to 21 kg); analyses adjusted for maternal characteristics

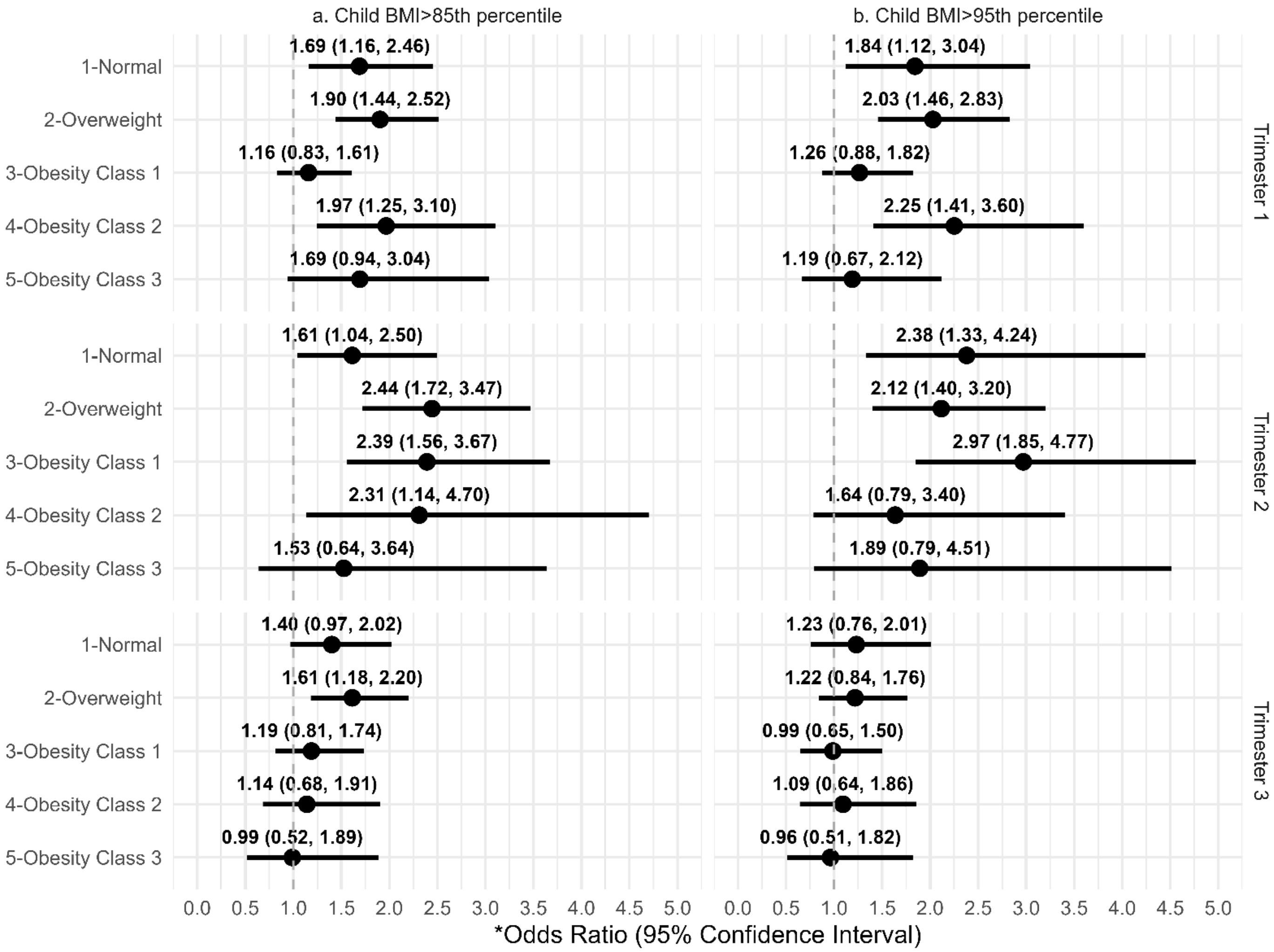

#### Figure 5

Relative odds* of child BMI (a) >85th percentile (overweight) and (b) >95th percentile (obese) according to rate of maternal trimester-specific gestational weight gain rate (kg/week)

*Represents the expected relative change in odds of child having BMI>85% associated with a 1kg/week unit increase in GWG (e.g., from 1 to 2 kg/week); analyses adjusted for previous trimester(s) GWG rate(s) and maternal characteristics

## Supplementary Files

This is a list of supplementary files associated with this preprint. Click to download.

- SupplementalFigure1.pdf

- SupplementalFigure2.pdf
- SupplementalFigure3.pdf
